# Mapping of a Novel Race Specific Resistance Gene to Phytophthora Root Rot of Pepper (*Capsicum annuum*) Using Bulked Segregant Analysis Combined with Specific Length Amplified Fragment Sequencing Strategy

**DOI:** 10.1371/journal.pone.0151401

**Published:** 2016-03-18

**Authors:** Xiaomei Xu, Juan Chao, Xueli Cheng, Rui Wang, Baojuan Sun, Hengming Wang, Shaobo Luo, Xiaowan Xu, Tingquan Wu, Ying Li

**Affiliations:** 1 Vegetable Research Institute, Guangdong Academy of Agricultural Sciences, Guangzhou, China; 2 Guangdong Key Lab for New Technology Research of Vegetables, Guangzhou, China; Agriculture and Agri-Food Canada, CANADA

## Abstract

Phytophthora root rot caused by *Phytophthora capsici* (*P*. *capsici*) is a serious limitation to pepper production in Southern China, with high temperature and humidity. Mapping PRR resistance genes can provide linked DNA markers for breeding PRR resistant varieties by molecular marker-assisted selection (MAS). Two BC_1_ populations and an F_2_ population derived from a cross between *P*. *capsici*-resistant accession, Criollo de Morelos 334 (CM334) and *P*. *capsici*-susceptible accession, New Mexico Capsicum Accession 10399 (NMCA10399) were used to investigate the genetic characteristics of PRR resistance. PRR resistance to isolate Byl4 (race 3) was controlled by a single dominant gene, *PhR10*, that was mapped to an interval of 16.39Mb at the end of the long arm of chromosome 10. Integration of bulked segregant analysis (BSA) and Specific Length Amplified Fragment sequencing (SLAF-seq) provided an efficient genetic mapping strategy. Ten polymorphic Simple Sequence Repeat (SSR) markers were found within this region and used to screen the genotypes of 636 BC_1_ plants, delimiting *PhR10* to a 2.57 Mb interval between markers P52-11-21 (1.5 cM away) and P52-11-41 (1.1 cM). A total of 163 genes were annotated within this region and 31 were predicted to be associated with disease resistance. *PhR10* is a novel race specific gene for PRR, and this paper describes linked SSR markers suitable for marker-assisted selection of PRR resistant varieties, also laying a foundation for cloning the resistance gene.

## Introduction

Phytophthora blight, caused by *Phytophthora capsici* (Leon.), is one of the most destructive pepper diseases worldwide [1]. Depending on the point of infection, *P*. *capsici* may cause several different disease syndromes, including root rot, stem rot, fruit rot and foliar blight in pepper [2]. Phytophthora root rot (PRR) of pepper, usually causing plant death, is the most serious pepper disease and may lead to total crop failure [3]. Currently, chemical control is the main management strategy for PRR, which not only increases production costs, but also results in environmental pollution. Utilization of resistant varieties is the most economical and environmentally friendly strategy to prevent this disease [4].

Host resistance, one form of disease resistance in plants, is characterized by specific plant varieties resisting infection by one or a few pathogenic races but being susceptible to other races [5]. Though the inheritance of resistance to *P*. *capsici* in pepper is complex and differs among plant populations, disease screening conditions and pathogen isolates, two distinct types of host resistance to *P*. *capsici* on pepper have been reported: (1) race-specific resistance controlled by a single dominant gene [2, 6, 7] and (2) partial resistance conferred by quantitative trait loci (QTLs) acting together [8–10].

Identification and mapping of resistance genes and QTLs conferring partial resistance to *P*. *capsici* in pepper is a prerequisite to MAS for *P*. *capsici* resistance. To date, almost one hundred QTLs for *P*. *capsici* resistance in pepper have been identified [8–15]. The majority of the QTLs have been identified on chromosome 5, and are considered to reflect a major effect locus that has been detected in all of the resistant pepper accessions studied to date [16]. Several types of linked markers for this major QTL have been developed, including Sequence Characterized Amplified Region (SCAR) and Randomly Amplified Polymorphic DNA (RAPD) markers [17, 18], Bacterial Artificial Chromosome (BAC)-derived markers [11] and recently developed SNP markers [19]. These diagnostic markers offer promise toward MAS for *P*. *capsici* resistance in pepper. In contrast to several recent reports of QTLs conferring partial resistance to *P*. *capsici*, no race-specific resistance gene has been identified, although resistance against *P*. *capsici* in pepper had been reported to be controlled by a single dominant gene [2, 6, 7]. Interestingly, a novel gene that inhibits resistance to *P*. *capsici* (*Ipcr*) was found in a *P*. *capsici*-susceptible accession, NMCA10399 [20]. When the most *P*. *capsici*-resistant accession, CM334 [21], was hybridized with NMCA10399, the resultant F_1_ populations were completely susceptible and the F_2_ displayed a 3:13 (R: S) segregation ratio to *P*. *capsici*-induced root rot and foliar blight disease, indicating that the *Ipcr* gene has an epistatic dominant effect over the dominant resistance genes for root rot and foliar blight.

Bulked segregant analysis (BSA), first proposed by Michelmore et al. [22], has been used for more than 20 years to identify molecular markers linked to a target gene or major QTL affecting a trait of interest [23–25]. BSA is a rapid and effective method that involves genotyping only two bulked DNA samples, respectively from groups of individuals with distinct phenotypes (for example, resistant and susceptible). However, it is a challenge for researchers to develop thousands of candidate molecular markers to screen the pools to find a small subset of markers diagnostic of the target phenotype.

A newly developed technology, SLAF-seq, combines high-throughput and reduced representation library (RRL) sequencing, offering an efficient and high-resolution strategy for genome-wide genotyping [26]. SLAF-seq has been used for genetic map construction and QTL analysis in sesame [27], soybean [28, 29] and cucumber [30, 31]. New strategies have recently been proposed to take advantage of the power of both BSA and SLAF-seq technologies. Two examples have been reported, delineating a maize inflorescence meristem mutant to a region of 3.947 Mb [32], and identifying major QTLs associated with rice grain weight [33].

In the present study, we aimed to (1) investigate the inheritance mode of PRR resistance against a specific race of *P*. *capsici*, (2) find PRR resistance gene-containing regions by integrating BSA with SLAF-seq technology, (3) develop SSR markers and segregating populations to carry out linkage analysis and narrow down the size of the gene-containing regions, providing diagnostic SSR markers for MAS of PRR resistant varieties and laying a foundation for cloning the resistance gene.

## Materials and Methods

### Plant materials and growth conditions

CM334, a landrace from Mexico, with the highest known resistance to *P*. *capsici*, was used as resistant parent (female) and NMCA10399, a *P*. *capsici*-susceptible accession [20], was used as susceptible parent (male). Derived from CM334 and NMCA10399, two BC_1_ populations, (CM334×NMCA10399)×CM334, comprising 222 plants and (CM334×NMCA10399)×NMCA10399, of 372 individuals, respectively, and an CM334×NMCA10399 F_2_ population of 259 plants were used for genetic analysis. After genetic analysis of PRR resistance, a third BC_1_ population [(CM334×NMCA10399)×NMCA10399] of 436 individuals was then used to screen for PRR-resistant or PRR-susceptible plants to construct a resistant DNA pool (R-pool) and a susceptible DNA pool (S-pool). According to the result of marker-trait association, a fourth BC_1_ population [(CM334×NMCA10399)×NMCA10399] of 636 individuals was genotyped and phenotyped to finely map the PRR resistance gene. All plant materials were grown in 32-cell plastic trays filled with plant growth medium (Floragard, Germany). Three seeds per cell were sown. Seedlings were watered twice a day and kept in a greenhouse in which *P*. *capsici* is not normally encountered, at 25±5 and 18±5°C day and night temperatures, respectively, with a 12-h photoperiod. Plants were kept in the greenhouse until the four-to-six true leaf stage before treatment with *P*. *capsici*.

### Phenotype screening of PRR resistance

*P*. *capsici* isolate Byl4 was used as inoculum for PRR resistance screening. Byl4, which is identified as race 3 (data not show) was isolated from infected pepper plants in 2012 in Baiyun field, Guangzhou, Guangdong province, China. To screen for PRR reaction, plants at the four-to-six true leaf stage were tested as described by Bosland and Lindsey [3] with modifications. The plants grown individually in 32-cell plastic trays were watered 12h before inoculation, and then the soil of each cell was injected with 5 ml inoculum, counted with Bright-Line^™^ Hemacytometer (Hausser Scientific, USA), in a concentration of 1 × 10^4^ zoospores/ml. The treated plants were kept in a controlled plant growth chamber (RTOP-1000Y), at 25°C in darkness for 24h with 80% relative humidity, and then kept at 25°C with 14-h light and 10-h dark cycle. The plants were scored for PRR when the stem of susceptible control NMCA10399 become tan or slightly darkened at the soil line, about 3 days after treatment. We scored once a day until all susceptible control plants showed symptoms (about 10 days after treatment). The plants with no symptoms were considered resistant, while plants showing symptoms ranging from slight root-stem darkening to death were all considered susceptible.

### DNA extraction and pool construction

Total genomic DNA was isolated from young leaves of parents and BC_1_ plants using a CTAB (cetyl trimethylammonium bromide) method with minor modifications [34]. DNA was quantified using a NanoDrop 2000 spectrophotometer (Thermo Scientific, USA). Fifty PRR resistant plants and fifty susceptible plants were selected from the BC_1_ population of 436 individuals and equal amounts of DNA from each plant were mixed to form the R-pool and S-pool, respectively, at a final concentration of 40 ng/μl.

### SLAF library construction and sequencing

The present study used the reference genome of CM334, with an assembled size of 3.06 Gb (http://peppergenome.snu.ac.kr/, V. 1.55). A simulated restriction enzyme digestion was carried out to establish conditions to optimize SLAF yield, avoid repetitive SLAFs, and obtain an even distribution of SLAFs for maximum SLAF-seq efficiency. Based on the results of this preliminary experiment, genomic DNA of two parents and pools were digested with the *Hae*III restriction enzyme, and *Arabidopsis thaliana* was used as control to assess the effectiveness of enzyme digestion. The SLAF libraries were constructed according to procedures described by Sun et al. [26]. The average SLAF sequencing depth should be at least 10x for each parent to cover all SNP sites, and 1x for each progeny (50 progenies for each pool) to eliminate background noises. To obtain higher coverage for pools, more samples of pool libraries should be added in the final solution than of the parents when sequenced. DNA fragments of 314-364bp were selected as SLAFs and prepared for paired-end sequencing on an Illumina High-seq 2500 platform (Illumina, Inc.; San Diego, CA, US) at Beijing Biomarker Technologies Corporation (http://www.biomarker.com.cn).

### Sequence data analysis and SLAF definition

Raw sequence reads (101bp in length) were filtered and trimmed for quality and adaptor removal, with 80-bp paired sequences retained at each end. The trimmed reads were then clustered based on sequence similarity (90% identity) among the four libraries (two parents and two pools) by BLAST as described by Kent [35]. All SLAFs were used to estimate sequence depth of parents and pools. SLAFs were defined based on parental sequences and pools were genotyped based on similarity to parental sequences. As a diploid species, pepper SLAFs with more than 4 tags were defined as repetitive SLAFs, those with only 1 tag were defined as monomorphic SLAFs, and those with 2–4 tags were polymorphic SLAFs. High-quality polymorphic SLAFs for which parents are both homozygous and with a summed sequence depth more than 5 fold were used for association studies.

### Association analysis

The SNP-index algorithm is a method useful to find significant differences of genotype frequency between DNA pools. As an SNP-index method [36], we used SLAF depth within a DNA pool as genotype frequency. The Δ(SNP-index) was calculated based on SNP-index (_Rp_) = S_Rp_/(S_Rp_+R_Rp_), and SNP-index (_Sp_) = S_Sp_/(S_Sp_+R_Sp_), with Δ(SNP-index) = SNP-index (_Sp_)- SNP-index (_Rp_). R_Rp_ and S_Rp_ represent the depth of the R-pool derived from resistant and susceptible parents, respectively; and R_Sp_ and S_Sp_ indicate the depth of the S-pool derived from resistant and susceptible parents, respectively. As we used a BC_1_ population for mapping, the upper limit Δ(SNP-index) of the trait—associated SLAFs is expected to be 0.5. We carried out Loess regression fitting to determine and obtain the threshold for significance of marker-trait associations as described by Hill et al. [37]. The regions over the threshold were considered as trait related candidate regions.

### SSR marker development and genotyping

The genome sequences of the PRR resistance related regions were downloaded from the CM334 reference genome (http://peppergenome.snu.ac.kr/). SSRHunter 1.3 [38] was used to find SSR sites and Primer Premier 5.0 (Premier Biosoft International) was used to design SSR primers. All SSR markers were screened for polymorphisms among parents and the two DNA pools. SSR markers which were polymorphic between both the two parents and the two pools were used to genotype the BC_1_ population of 636 individuals. PCR was performed as described by Piquemal et al. [39] with minor modifications: 94°C for 5 min; 8 cycles of 94°C for 35 s, 57°C for 30 s and 72°C for 30 s; followed by 8 cycles of 94°C for 35 s, 53°C for 30 s and 72°C for 30 s; then 20 cycles of 94°C for 35 s, 55°C for 30 s and 72°C for 30 s, and a final incubation at 72°C for 7 min. PCR products were separated on an 8% polyacrylamide denaturing sequencing gel and visualized by silver nitrate staining.

### Genetic analysis and linkage map construction

To determine the mode of inheritance of PRR resistance, Chi-squared (χ^2^) analysis was carried out to test the phenotypic data for goodness-of-fit to Mendelian segregation ratios. Using the phenotype and genotype data from the 636 BC_1_ plants, a linkage map was constructed with Mapmaker/EXP 3.0 [40], using a LOD threshold of 3.0.

## Results

### Genetic analysis of PRR resistance

**Fig 1:**
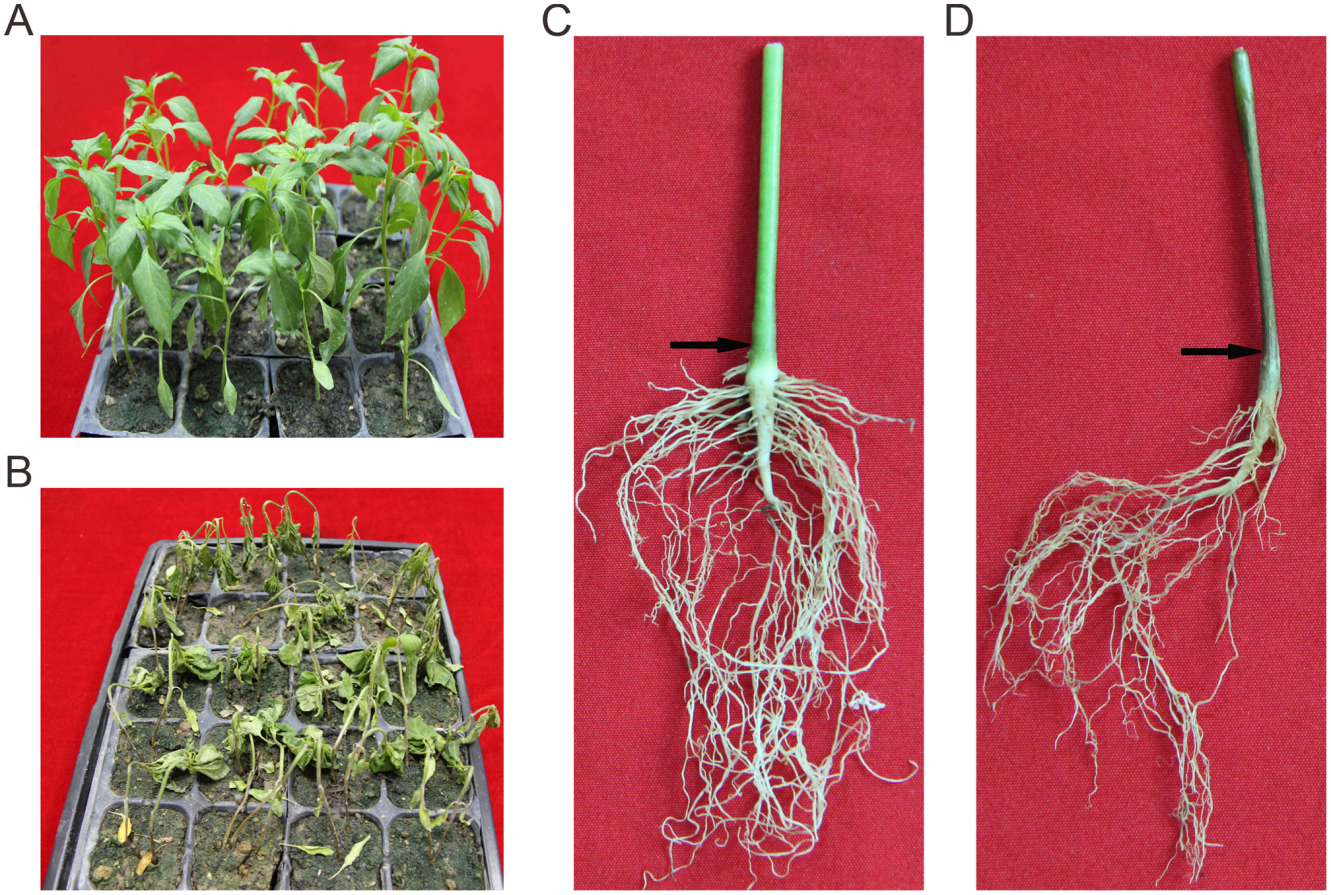
Symptoms for *P*. *capsici* induced root rot of pepper. A, The phenotype of CM334 9 days post-inoculation. B, The phenotype of NMCA10399 9 days post-inoculation. C, Roots of CM334 9 days post-inoculation. D, Roots of NMCA10399 9 days post-inoculation. The arrows indicate the stem at the soil line.

Two BC_1_ populations and an F_2_ population derived from a cross between CM334 and NMCA10399 were used to investigate the genetic characteristics of PRR resistance. In phenotype screening experiments, all 41 plants of the resistant parent (CM334) showed resistance with no lesions observed, while the 66 plants of the susceptible parent (NMCA10399) were all dead 9 days after treatment (Fig 1). The backcross population, (CM334×NMCA10399)×CM334, comprising 222 plants, all displayed resistance. Among the other backcross population, (CM334×NMCA10399)×NMCA10399, of 372 individuals, 177 plants showed resistance and 195 were susceptible, values which did not deviate significantly from a 1:1 segregation ratio (χ^2^ = 0.78, P > 0.05). Of the 259 F_2_ plants, 190 were resistant and 69 were susceptible, with a 2.75:1 segregation ratio that did not deviate significantly from a 3:1 ratio (χ^2^ = 0.29, P > 0.05). Each of these results indicated that PRR resistance to isolate Byl4 was controlled by a single dominant gene.

### SLAF-seq data analysis and SLAF identification

Four samples yielded a total of 74.79 million paired-end reads with 80-bp valid read length, and an average Q30 (error rate of 0.1% per base) ratio of 86.1% (Table 1). After clustering, 250,871 SLAFs were procured. The average sequence depths of SLAFs were ~34.0 and ~23.1 fold in resistant (CM334) and susceptible parent (NMCA10399), respectively; and ~64.1 and ~58.8 fold in the R-pool and S-pool, respectively (Table 1). Mapping these SLAFs on the reference genome of CM334, we calculated the SLAF numbers on each chromosome (Table 2) and could see SLAFs distributing evenly on chromosomes (S1 Fig), suggesting good representation of the genome. These SLAFs were divided into three types: 77.94% (195,534) monomorphic, 1.77% (4,454) repetitive, and 20.28% (50,883) polymorphic. A total of 42,036 polymorphic SLAFs (Table 2, S2 Fig) for which parents are both homozygous and with a summed sequence depth more than 5 fold, were used to do marker-trait association studies.

**Table 1:** Summary of sequencing data for parental lines and DNA pools.

Sample ID	Total reads	Q30 (%)	Average depth
CM334	12,982,436	88.14	33.99
NMCA10399	9,634,836	87.65	23.06
R-pool	26,780,391	85.84	64.06
S-pool	25,399,611	85.09	58.79

**Table 2:** Distribution of SLAFs, polymorphic SLAFs and high-quality polymorphic SLAFs over the pepper chromosomes.

Chr ID	SLAFs	Polymorphic SLAFs	High-quality polymorphic SLAF
Chr01	23,307	5,042	4,419
Chr02	21,637	2,595	2,236
Chr03	21,147	5,445	4,794
Chr04	23,822	2,673	2,272
Chr05	15,038	4,377	3,570
Chr06	14,196	6,297	5,562
Chr07	24,149	5,019	4,441
Chr08	21,568	1,750	1,281
Chr09	21,327	4,626	4,045
Chr10	20,991	2,941	2,468
Chr11	20,761	7,413	4,742
Chr12	22,928	2,705	2,206
Total	250,871	50,883	42,036

See ‘Materials and methods’ for the definition of ‘SLAFs’, ‘polymorphic SLAFs’ and ‘high-quality polymorphic SLAFs’.

### Marker-trait associations

**Fig 2:**
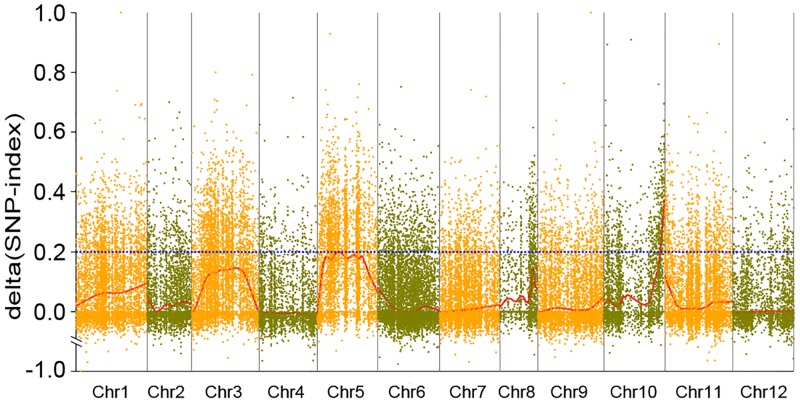
Graph of Δ(SNP-index) from analysis of SLAF-seq marker-trait association. The *X*-axis represents chromosomal position and the *Y*-axis represents Δ(SNP-index). The blue dashed line indicates the association-threshold. A region related to PRR resistance was identified in pepper chromosome 10 (217.17–233.56 Mb interval) where the Δ(SNP-index) consistently exceeded the association-threshold. Higher Δ(SNP-index) indicates stronger association.

For the high-quality polymorphic SLAFs, SNP-index (_Rp_) and SNP-index (_Sp_) were calculated, used as described to determine Δ(SNP-index), and mapped to their position in the CM334 reference genome. To locate trait related candidate regions, we carried out Loess regression fitting of the Δ(SNP-index) resulting from these SLAFs. The marker-trait association threshold was 0.19 after Loess regression fitting and a total of 7,526 SLAFs exceeding the threshold level, suggesting significant differences between the pools. As a result, we found an interval of 217.17–233.56 (16.39) Mb at the end of the long arm of chromosome 10 with one hundred and thirty-nine SLAF markers consistently exceeding the association-threshold (Fig 2). In this interval, a sub-region containing three consecutive SLAFs with SNP-index (Sp) of 1 was found, suggesting the *PhR10* gene may be in or very near it.

### Validation and mapping

**Fig 3:**
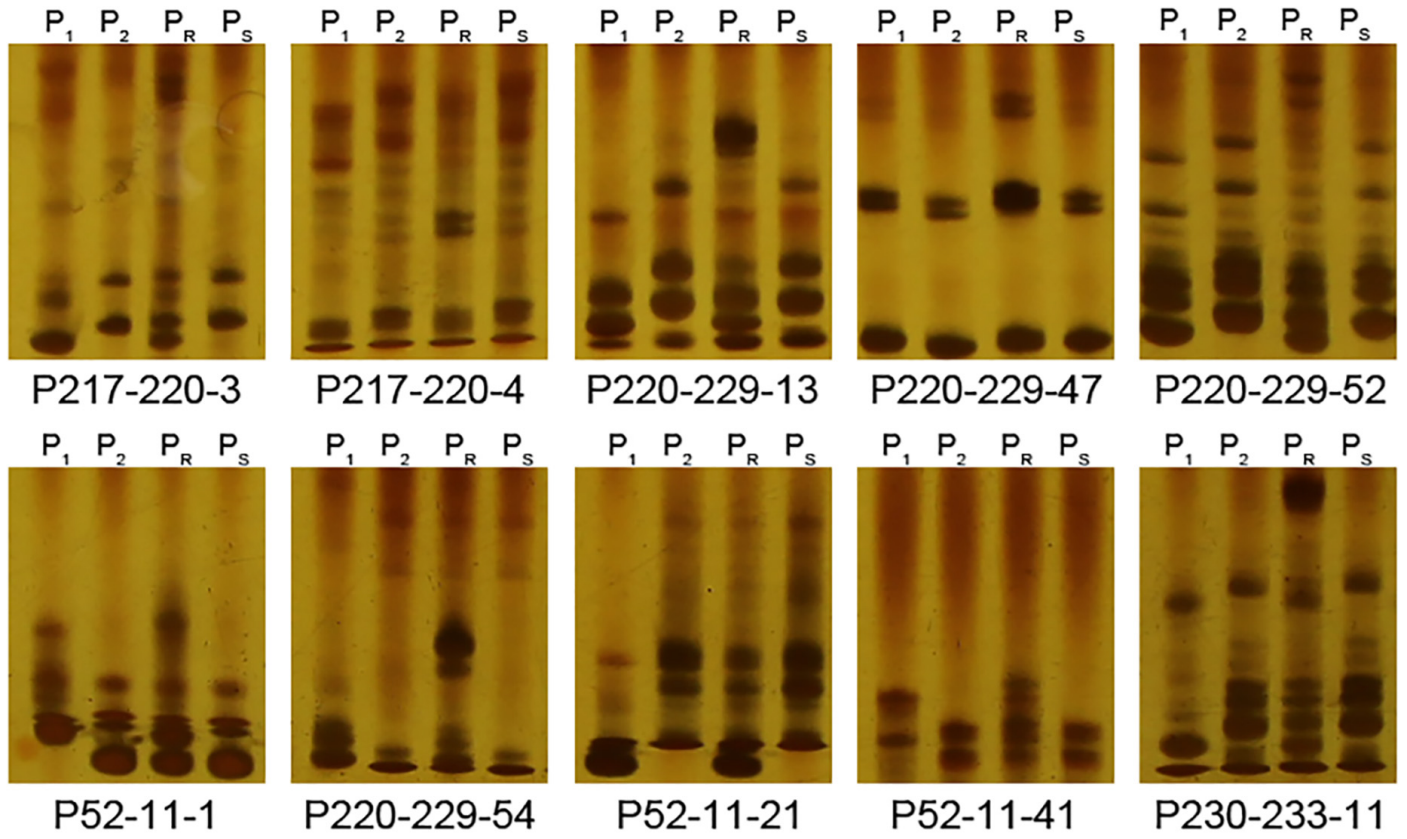
Gel images of 10 polymorphic SSR markers in parents and pools. P_1_, P_2_, P_R_ and P_S_ represent the resistant parent CM334, susceptible parent NMCA10399, resistant pool and susceptible pool, respectively.

**Fig 4:**
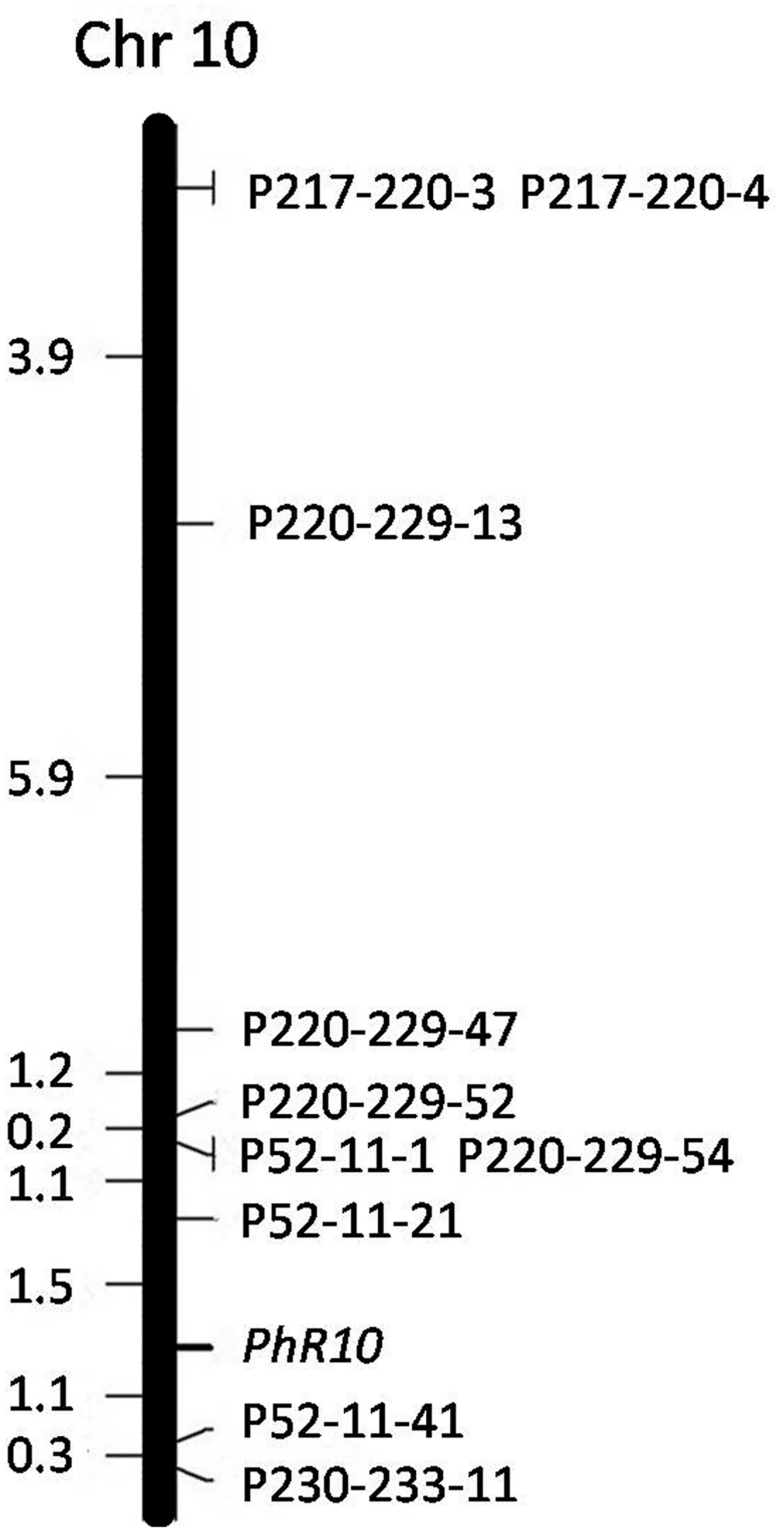
Genetic linkage map of *PhR10* constructed from the BC_1_ population of (CM334×NMCA10399)×NMCA10399. Map distance in Kosambi centiMorgans (cM) is on the left, SSR markers are arranged on the right.

The sequences of the PRR resistance related region (16.39 Mb) were downloaded from the CM334 reference genome (http://peppergenome.snu.ac.kr/). A total of 197 Simple Sequence Repeat (SSR) sites were found in the region by using the SSRHunter 1.3 software. Primers were designed and screened for polymorphisms between both the parents and pools. Two SSR markers polymorphic between the parents but not the pools were not used in further analysis. Only 10 SSR markers (S1 Table) were polymorphic between both the parents and the pools with heterozygous amplicons in R-pool and homozygous amplicons the same as NMCA10399 in S-pool (Fig 3), suggesting the marker and trait are associated, which confirmed the association analysis was reliable. These 10 markers were used to screen the genotypes of 636 BC_1_ plants used for genetic linkage analysis. The trait-related locus *PhR10* was flanked by SSR markers P52-11-21 and P52-11-41 at distance of 1.5 and 1.1 cM, respectively (Fig 4).

### Gene annotation and candidate gene prediction

SSR markers P52-11-21 and P52-11-41 are at positions 229,191,632 and 231,757,882, respectively, delineating approximately 2.57Mb of DNA that contains *PhR10*. According to a pepper gene annotation database (http://peppergenome.snu.ac.kr/, V. 1.55), 163 genes were annotated within this region. Among these genes, 31 were predicted to be associated with disease resistance (S2 Table).

## Discussion

The genome size of pepper is large, estimated to be 3.48 Gb [41]. Whole genome deep re-sequencing or low coverage sequencing is relatively costly for large genomes and usually unnecessary for gene/QTL mapping. RRL sequencing is one strategy to bring down the cost by genome reduction, sampling and sequencing a small subset of genomic segments instead of the whole genome [42]. SLAF-seq is a recently developed enhanced RRL sequencing strategy for genome-wide SNP discovery and has been used for linkage map construction and QTL analysis in organisms such as sesame [27], soybean [28, 29] and cucumber [30, 31]. In this study, we used BSA combined with SLAF-seq strategy to do preliminary mapping of PRR resistance gene, generating a total of 74.79 million paired-end reads with 80-bp valid read length, developing 50,883 (20.28%) polymorphic SLAFs with the proportion of polymorphic SLAFs ranging from 8.11% (1,750 of 21,568) on chromosome 8 to 44.36% (6,297 of 14,196) on chromosome 6 (Table 2), suggesting biased distribution of polymorphic markers (SLAFs) among chromosomes. This conforms to other result that chromosome 8 showed the lowest density of SNP distribution [41], indicating a relatively low evolution pressure in it. Finally, 42,036 high-quality polymorphic SLAFs were used to do marker-trait association analysis and a PRR resistance related region of 16.39Mb was found at the end of the long arm of chromosome 10. This showed that BSA combined with SLAF-seq is a rapid and effective method for initial gene mapping and laid a foundation for gene fine mapping.

To date, no race-specific resistance genes had been identified, although resistance against *P*. *capsici* in pepper had been reported to be controlled by a single dominant gene [2, 6, 7]. Results in this study showed that under the inoculation of race 3, which was the dominant physiological race of *P*. *capsici* on pepper in Guangdong province of China [43], a dominant PRR resistance gene *PhR10* was identified and mapped on chromosome 10. However, interestingly, a novel gene *Ipcr* having an epistatic dominant effect over the dominant resistance genes for root rot and foliar blight was found by using genetic populations derived from CM334 and NMCA10399 [20], which were the same parents used in our study. At first view, it sounds inconceivable, as the methods of phenotype screening of PRR resistance were also almost the same, only with a difference in final concentration of inoculum, 5× 10^4^ zoospores/plant in our study and 1 × 10^4^ zoospores/plant in that. In addition, to test the effect of *Ipcr* on race-specific resistance, *P*. *capsici* races -*1* (American Type culture Collection, ATCC: MYA-2289), -*2* (ATCC: MYA-2291), and -*12* (not in ATCC) isolated from *C*. *annuum* in New Mexico and race-*15* (ATCC: MYA-2339) isolated from *C*. *annuum* in New Jersey were used to inoculate populations for root rot and foliar blight. Results showed that *Ipcr* had an epistatic dominant effect over the dominant resistance genes for root rot when treated with four races (race -*1*, race -*2*, race -*12* and race -*15*) and only one race (races -*1*) for foliar blight, indicating that *Ipcr* interferes with tissue- and race-specific resistance for *P*. *capsici*. Therefore, we inferred that the different physiological races of *P*. *capsici* used in these two studies are the causes of different results.

The number and position of *P*. *capsici* resistance QTLs identified in a study vary depending on populations used, disease screening conditions, traits measured to reflect disease, and isolates of *P*. *capsici*. Almost one hundred QTLs for *P*. *capsici* resistance in pepper have been identified [8–15], with a consistently identified QTL on chromosome 5 considered to be a major QTL for resistance to *P*. *capsici* [16, 19]. Most other previously reported QTLs were located on chromosomes 6, 9, 11 and 12 [44]. Thabuis et al. [9] performed QTL analysis by using three different intraspecific pepper populations. Two QTLs for PRR resistance were consistent in two populations, located at the end of the short arm of chromosome 10. Additionally, three isolate-specific QTLs associated with PRR resistance were detected in the middle of chromosome 10 and another was found at the end of the short arm of chromosome 10 [8]. In the present study, a PRR resistance to *P*. *capsici* race 3 related region at the end of the long arm of chromosome 10 [217.17–233.56 (16.39) Mb] was detected by a strategy combining BSA with SLAF-seq technology, and was verified by classical genetic mapping. The location of this PRR race specific resistance gene is clearly different from previously identified QTLs on chromosome 10.

Breeding resistant cultivars remains the most effective strategy to reduce losses caused by *P*. *capsici*. Physiological race differentiation exists in the *P*. *capsici*- *C*. *annuum* interaction [45, 46] and different genes confer resistance to different physiological races of *P*. *capsici* [6]. It may be impossible to breed a resistant cultivar with universal resistance to all *P*. *capsici* races on pepper, but it may be possible to breed for resistance to isolates found in specific growing regions. In Guangdong province of China, the dominant physiological race of *P*. *capsici* on pepper is race 3 [43]. In this study, we identified a locus, *PhR10*, at which one allele confers resistance to *P*. *capsici* isolates Byl4 which was identified as race 3. The flanking SSR markers P52-11-21 and P52-11-41, respectively 1.5 and 1.1 cM from *PhR10*, will be convenient tools for MAS of PRR resistance in pepper breeding program.

## Supporting Information

S1 Fig Distribution of total SLAFs on chromosomes. (TIF)

S2 Fig Distribution of high-quality polymorphic SLAFs on chromosomes. (TIF)

S1 Table Description of 10 polymorphic SSR markers. (DOCX)

S2 Table Annotated genes associated with disease resistance. (XLSX)
