# Supplementary figures and images for: Mapping of a Novel Race Specific Resistance Gene to Phytophthora Root Rot of Pepper (Capsicum annuum) Using Bulked Segregant Analysis Combined with Specific Length Amplified Fragment Sequencing Strategy
Source: PLoS One. 2016 Mar 18;11(3):e0151401. doi: 10.1371/journal.pone.0151401 (PMC4798474; doi:10.1371/journal.pone.0151401)

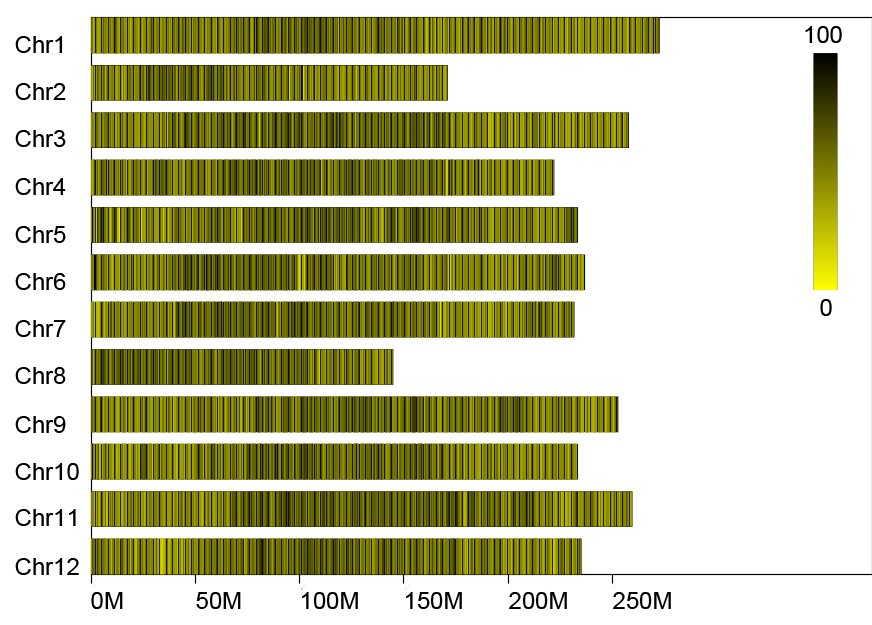

Supplement: S1 Fig — (TIF) [file pone.0151401.s001.tif]

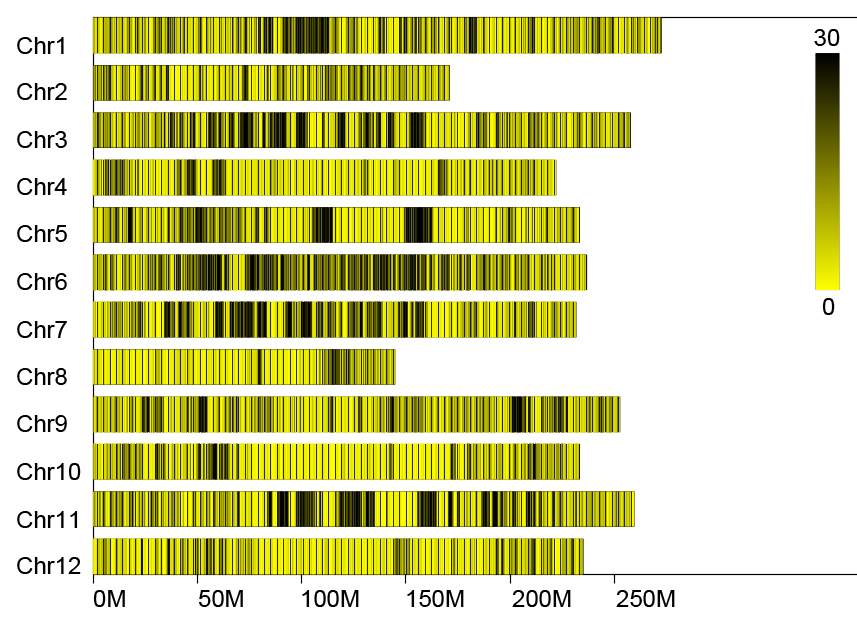

Supplement: S2 Fig — (TIF) [file pone.0151401.s002.tif]
