# Supplementary material for: Mapping of a Novel Race Specific Resistance Gene to Phytophthora Root Rot of Pepper (Capsicum annuum) Using Bulked Segregant Analysis Combined with Specific Length Amplified Fragment Sequencing Strategy
Source: PLoS One. 2016 Mar 18;11(3):e0151401. doi: 10.1371/journal.pone.0151401 (PMC4798474; doi:10.1371/journal.pone.0151401)
Supplement: S1 Table — (DOCX) [file pone.0151401.s003.docx]

S1 Table Description of 10 polymorphic SSR markers.

| Marker | Primer sequences (5' ^_^3') | Position (bp) |
| --- | --- | --- |
| P217-220-3 | F: GAGTAAACCGATAATCCAAT | 217,487,356 |
|  | R: ATGTTAGTTAGGAGGAATTA |  |
| P217-220-4 | F: TTCCTTTATGTCTAGGCTTT | 217,509,093 |
|  | R: CAGTTTTCAGGTACATTACT |  |
| P220-229-13 | F: TGTTTGTGATTTTTTGTGGG | 221,349,928 |
|  | R: AATGAGGAGACGATTTGTAT |  |
| P220-229-47 | F: AAGAAAAATGAACCTACGAG | 227,031,752 |
|  | R: ACCACCCCTATATAAAGATC |  |
| P220-229-52 | F: TCCATAACCAGAAAAGCATT | 228,043,394 |
|  | R: AGCCTCTTCATCTGAGCATC |  |
| P52-11-1 | F: GATACTTTAGGGTTAATGGG | 228,308,777 |
|  | R: CAGAGGGTTTCATTCTTATT |  |
| P220-229-54 | F: TAATGGGGTTCAACATCTAC | 228,308,790 |
|  | R: CTTTTTGTTCCTTATCACTT |  |
| P52-11-21 | F: CAATCCAAACAAGTCCTAAG | 229,191,632 |
|  | R: GGTGCAATTGAAAATCTAAG |  |
| P52-11-41 | F: TTGATGAGATGGGAAGTAAA | 231,757,882 |
|  | R: CACCAACAATAATAGAACTACA | |
| P230-233-11 | F: ATAGAATGACTTCCAGGCAA | 232,062,165 |
|  | R:AAAGGTAAGGAGTAAGGCTG |  |
